# Clinical Effectiveness and Safety of Reduced-Dose Prasugrel in Asian Patients: The PROMISE-TW Registry

**DOI:** 10.3390/jcm14165791

**Published:** 2025-08-15

**Authors:** Yu-Chen Wang, Chiung-Ray Lu, Yi-Tzone Shiao, Kuan-Cheng Chang, Chun-Hung Su, Yu-Wei Chiu, Chien-Lung Huang, Wei-Shin Liu, Ching-Lung Yu, Ming-Jer Hsieh, Ye-Hsu Lu, Ho-Ming Su, Po-Chih Lin, Hsin-Bang Leu, Wen-Lieng Lee

**Affiliations:** 1 Division of Cardiology, Department of Internal Medicine, Asia University Hospital, No. 222, Fuxin Rd., Wufeng Dist., Taichung 413505, Taiwan; 2 Department of Medical Laboratory Science and Biotechnology, Asia University, Taichung 41354, Taiwan; 3 Division of Cardiovascular Medicine, Department of Internal Medicine, China Medical University Hospital, Taichung 404327, Taiwan; ha02410@everanhospital.com.tw (C.-R.L.); kuancheng.chang@gmail.com (K.-C.C.); 4 School of Medicine, China Medical University, Taichung 406040, Taiwan; 5 Department of General Education, Chihlee University of Technology, New Taipei City 22050, Taiwan; ytshiao@gmail.com; 6 Department of Internal Medicine, Chung Shan Medical University Hospital, Taichung 402306, Taiwan; such197408@gmail.com; 7 Department of Internal Medicine, School of Medicine, Chung Shan Medical University, Taichung 40201, Taiwan; 8 Cardiology Division, Cardiovascular Medical Center, Far Eastern Memorial Hospital, New Taipei City 220216, Taiwan; dtmed005@yahoo.com.tw; 9 Department of Computer Science and Engineering, Yuan Ze University, Taoyuan 320314, Taiwan; 10 Division of Cardiology, Heart Center, Cheng Hsin General Hospital, Taipei 112401, Taiwan; clhuang0130@gmail.com; 11 School of Medicine, College of Medicine, National Yang Ming Chiao Tung University, Taipei 11221, Taiwan; 12 Division of Cardiology, Buddhist Tzu Chi Medical Foundation, Hualien Tzu Chi Hospital, Hualien 970473, Taiwan; wsliu22@yahoo.com.tw; 13 Division of Cardiology, Tainan Municipal Hospital, Tainan 709, Taiwan; yucl0116@gmail.com; 14 Division of Cardiology, Department of Internal Medicine, Chang Gung Memorial Hospital, Linkou 33305, Taiwan; mjhptcastent@gmail.com; 15 College of Medicine, Chang Gung University, Taoyuan 33302, Taiwan; 16 Division of Cardiology, Department of Internal Medicine, Kaohsiung Medical University Hospital, Kaohsiung 80708, Taiwan; yehslu@gmail.com; 17 Department of Internal Medicine, Kaohsiung Municipal Siaogang Hospital, Kaohsiung 807378, Taiwan; cobeshm@seed.net.tw; 18 Division of Cardiology, Department of Internal Medicine, National Taiwan University Hospital, Taipei 100225, Taiwan; juipeter@ntuh.gov.tw; 19 Cardiovascular Research Center, National Yang Ming Chiao Tung University, Taipei 10002, Taiwan; hbleu@vghtpe.gov.tw; 20 Healthcare and Management Center, Taipei Veterans General Hospital, Taipei 11217, Taiwan; 21 Division of Cardiology, Department of Internal Medicine, Taipei Veterans General Hospital, Taipei 11217, Taiwan; 22 Cardiovascular Center, Taichung Veterans General Hospital, Taichung 407219, Taiwan; wllee@vghtc.gov.tw; 23 School of Medicine, National Chung Hsing University, Taichung 40227, Taiwan

**Keywords:** prasugrel, acute coronary syndrome, chronic coronary syndrome, antiplatelet therapy, PROMISE-TW registry, Taiwan

## Abstract

**Background:** Reduced-dose prasugrel is widely used in East Asia for acute coronary syndrome (ACS), but real-world data in diverse Asian populations are limited. This study evaluated its effectiveness and safety in Taiwanese patients. **Methods:** The PROMISE-TW Registry was a multicenter, retrospective study including 1167 patients with ACS or chronic coronary syndrome (CCS) treated with reduced-dose prasugrel (20 mg loading, 3.75 mg maintenance) across 13 hospitals in Taiwan from 2018 to 2022. The primary endpoint was 1-year major adverse cardiovascular events (MACEs: cardiovascular death, non-fatal myocardial infarction, and non-fatal stroke). Secondary outcomes included composite ischemic events and major bleeding (BARC 3–5). **Results:** Among enrolled patients (mean age 63.9 years; 81.2% male; 83% ACS), percutaneous coronary intervention was performed in 90.8%. At one year, MACEs occurred in 1.9%, composite ischemic events in 8.2%, and major bleeding in 0.8%. Subgroup analysis identified prior stroke, diabetes, and chronic total occlusion intervention as predictors of bleeding. Male sex, chronic kidney disease, and left circumflex artery intervention predicted higher ischemic risk. **Conclusions:**Reduced-dose prasugrel provided effective ischemic protection and low bleeding rates in Taiwanese patients, especially those with ACS. These findings support the clinical utility of dose-adjusted prasugrel in East Asian populations and highlight the importance of individualized risk assessment.

## 1. Introduction

Dual antiplatelet therapy (DAPT), comprising aspirin and a P2Y12 inhibitor, is the cornerstone of secondary prevention in patients with acute coronary syndrome (ACS) undergoing percutaneous coronary intervention (PCI) [1,2,3]. Prasugrel and ticagrelor have demonstrated superior efficacy over clopidogrel in preventing thrombotic complications, but their higher bleeding risk necessitates careful patient selection [4,5]. Recognizing these concerns in East Asian populations, who have a higher propensity for bleeding events, Taiwan has adopted a reduced-dose prasugrel regimen (20 mg loading, 3.75 mg maintenance), similar to that used in Japan [6]. However, data about the real-world effectiveness and safety of this regimen show conflicting results and remain underexplored [7,8,9,10]. While global trials such as TRITON-TIMI 38 have established full-dose prasugrel’s efficacy, the applicability of reduced-dose prasugrel to Taiwanese patients remains uncertain. Given the variations in genetic polymorphisms affecting drug metabolism and the unique bleeding profile in East Asian populations, the Prasugrel Reduced-dose Observation for Measuring Improvement in Safety and Effectiveness in Taiwan (PROMISE-TW) Registry was designed to evaluate the clinical outcomes of reduced-dose prasugrel in Taiwanese patients, providing essential data to inform treatment decisions and guideline development. By investigating a real-world cohort in Taiwan, this study seeks to bridge this knowledge gap and offer insights that may refine antithrombotic strategies in the region.

## 2. Methods

### 2.1. Study Design and Population

PROMISE-TW is a multicenter, retrospective cohort study conducted across 13 hospitals in Taiwan. Eligible patients were ≥18 years old, diagnosed with ACS or chronic coronary syndrome (CCS), and received reduced-dose prasugrel between January 2018 and November 2022 [11]. For de novo users, follow-up began on the index ACS diagnosis date coincident with prasugrel start. For patients who switched from another P2Y_12_ inhibitor, follow-up and outcome ascertainment began on the date of prasugrel initiation. Since prasugrel use for CCS was off-label in Taiwan, the analyses in this subgroup are exploratory and hypothesis generating. Patients were included regardless of PCI status or concomitant oral anticoagulant (OAC) use. Those with incomplete clinical data were excluded. The definition of complex PCI includes any one of the following criteria: left main (LM) lesion, bifurcation lesion, stent length > 30 mm, chronic total occlusion (CTO) lesion, implantation of ≥3 stents, or SYNTAX score ≥ 33. The definition of a fragile patient group includes those who meet all three of the following criteria: age > 75 years, body weight < 50 kg, and impaired renal function with an estimated glomerular filtration rate (eGFR) < 50 mL/min/1.73 m^2^.

### 2.2. Clinical Outcomes

The primary outcome was the incidence of major adverse cardiovascular events (MACEs), including cardiovascular death, non-fatal myocardial infarction (MI), and non-fatal stroke within one year after receiving prasugrel. Secondary outcomes included 1-year composite ischemic events (MACE plus unplanned revascularization), all-cause mortality, stent thrombosis, unplanned revascularization, major bleeding defined as Bleeding Academic Research Consortium (BARC) type 3 to 5 bleeding, and the combined major and minor bleeding endpoint comprised BARC types 2, 3, 4, and 5 to capture both minor (type 2) and major bleeding events. Cardiovascular death was defined as death due to cardiac causes, including MI, heart failure (HF), or fatal arrhythmias. Unplanned revascularization was defined as any repeat revascularization procedure, including PCI or coronary artery bypass grafting (CABG), which was not scheduled as part of the initial treatment plan.

### 2.3. Data Collection

Data were retrospectively recorded using a standardized online case report form. Local study coordinators at each site will gather additional information by completing an electronic data capture form based on patient medical records. Clinical data will be extracted from both hospital and outpatient records, including demographic information, comorbidities, lifestyle factors, medications, echocardiographic findings, angiographic data, and clinical outcomes [11]. A detailed list of study variables is provided in Supplementary Table S1. This study was approved by the institutional review boards of all participating hospitals. As this was a retrospective analysis using de-identified clinical data, the requirement for written informed consent was waived by each institutional review board.

### 2.4. Calculation of Sample Size

This study is a retrospective analysis, and all patients meeting the specified inclusion criteria are eligible for enrollment. According to the Japanese PRASFIT-PRACTICE II study, the one-year incidence of MACEs following reduced-dose prasugrel treatment was 1.7% [8]. Additionally, the Taiwan Acute Coronary Syndrome Full Spectrum Registry (2008–2010) reported a one-year MACE rate of 12.7% in patients with ACS [12]. Given the advancements in clinical techniques and pharmacological therapies for ACS in recent years, we estimate that the one-year MACE incidence for the same patient population enrolled in Taiwan between 2018 and 2022 may be approximately 3.5%. For a non-inferiority study design with a type I error of 0.025 and 80% power, a minimum of 969 ACS patients undergoing interventional treatment is required. However, considering the real-world use of reduced-dose prasugrel in CCS populations within Taiwan’s clinical setting, we plan to increase the total sample size to 1100 patients.

### 2.5. Statistical Analysis

Continuous variables were first tested for normality using the Shapiro–Wilk test. Variables with a normal distribution are presented as the mean ± standard deviation (SD), whereas non-normally distributed variables are shown as the median (interquartile range, IQR). Categorical data were compared by using the Chi-square test or Fisher’s Exact test in two independent groups. Logistic regression analysis was used to determine the variables affecting prognosis. Upon completion of the univariable analyses, we select variables for the multivariable analysis. Any variable whose univariable test has a *p*-value < 0.25 is a candidate for the multivariable model along with all variables of know clinical importance. Once the variables have been identified, we begin with a model containing all of then selected variables. Model fit was assessed by the Hosmer–Lemeshow test (where sample size permitted), discrimination by C-statistic, and collinearity by variance-inflation factor (VIF) (Supplementary Table S4). For each logistic model, we calculated the events-per-variable ratio (EPV). For the major-bleeding outcome (EPV = 3), we conducted LASSO penalized regression and compared the AIC and C-statistic to the full logistic model; as no performance benefit was observed, we retained the full model (Supplementary Table S5). A 2-tailed *p* value < 0.05 was considered statistically significant. All analyses were performed using the SAS 9.4 statistical package (SAS Institute Inc., Cary, NC, USA).

## 3. Results

**Figure 1:**
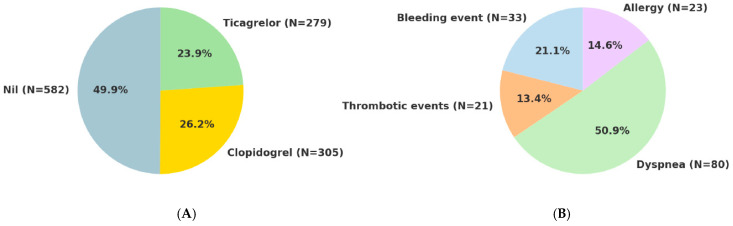
(**A**). The percentage of switching from other P2Y12 inhibitors to prasugrel. (**B**). Reasons for switching from other P2Y12 inhibitors to prasugrel.

This study enrolled 1167 patients with a mean age of 64.6 (IQR 15.5) years, and 13.9% were age older than 75. The majority of participants (81.2%) were male. Most patients (83%) presented with ACS, categorized as ST-segment elevation myocardial infarction (STEMI, 28.8%), non-ST-segment elevation myocardial infarction (NSTEMI, 26.9%), and unstable angina (23.0%), while the remaining 17% had CCS. DAPT including aspirin was prescribed to only 81.5% of patients during hospitalization. Regarding P2Y12 inhibitor therapy, approximately half (49.9%) received prasugrel as the initial medication; however, 26.2% switched from clopidogrel and 23.9% from ticagrelor. The primary reasons for switching were dyspnea (50.9%), bleeding events (21.1%), thrombotic events (13.4%), and allergies (14.6%) (Figure 1). Among patients who switched from a prior P2Y_12_ inhibitor to prasugrel, 63.5% (n = 671) did so within one month, 9.3% (n = 98) between 1 and 3 months, 4.2% (n = 44) between 3 and 6 months, 8.4% (n = 89) between 6 and 12 months, and 14.7% (n = 155) after more than 12 months. The overall duration of reduced-dose prasugrel therapy was 443.14 ± 329.02 days on average, with a median of 368 days (IQR 254).

PCI was performed in 90.8% of patients, with coronary stenting conducted in 80.6%, including drug-eluting stents in 69.7% and bare-metal stents in 13.4%. Complex PCI procedures were performed in 70.2% of cases, including PCI to the left main artery (8.3%), true bifurcation lesions (34.2%), CTO lesions (16.0%), use of stent lengths ≥ 30 mm (48.5%), implantation of three or more stents (10.5%), and SYNTAX scores ≥ 33 (6.3%).

In addition, 12.7% of patients required mechanical circulatory support, and atherectomy for severely calcified plaques was performed in 9.0% of cases. Radial artery access was used in 72.4% of interventions, while femoral access was employed in 22.3%. The target coronary vessels included the left main artery (8.3%), left anterior descending artery (LAD, 56.0%), left circumflex artery (LCX, 28.7%), and right coronary artery (RCA, 32.0%). Three-vessel coronary artery disease (CAD) was identified in 27.9% of patients.

The most common comorbidities were hypertension (HTN, 60.8%), diabetes mellitus (DM, 38.2%), hyperlipidemia (27.4%), chronic kidney disease (CKD, eGFR < 60, 19.0%), and a history of stroke (4.2%). Only 0.7% of the study population were classified as clinically fragile (Table 1).

**Figure 2:**
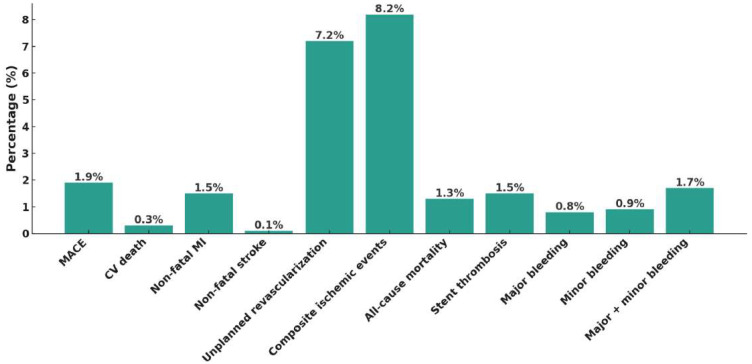
One-year clinical outcomes. The incidences of major adverse cardiovascular events (MACEs), composite ischemic events, stent thrombosis, major bleeding, and overall bleeding (major + minor) are shown. The overall safety and efficacy profile of reduced-dose prasugrel was favorable.

**Figure 3:**
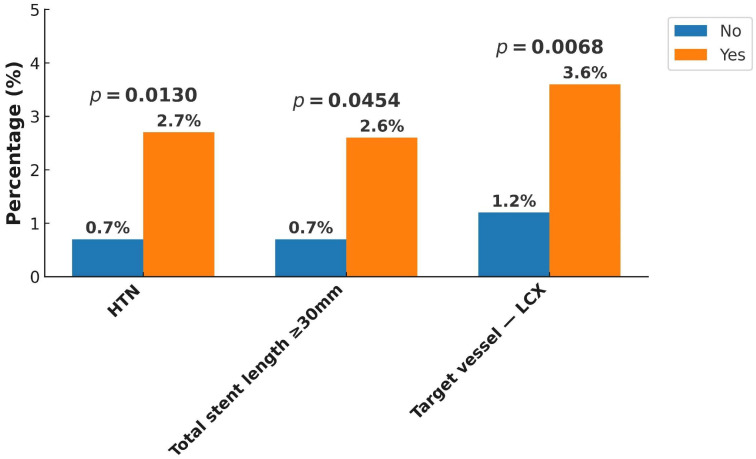
Subgroup analysis of one-year major adverse cardiovascular events (MACEs). A MACE was defined as cardiovascular death, non-fatal myocardial infarction (MI), or non-fatal stroke. Clinical characteristics such as hypertension, stent length ≥ 30 mm, and LCX as the target vessel were associated with higher MACE rates in exploratory analysis. Abbreviations: HTN, hypertension; LCX, left circumflex artery.

**Figure 4:**
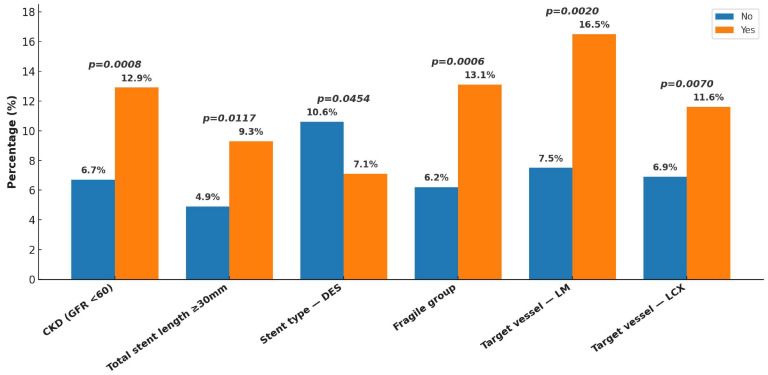
Subgroup analysis of one-year composite ischemic events. Composite ischemic events were defined as MACE plus unplanned revascularization. Factors such as CKD (eGFR < 60), long stent length, DES use, LM and LCX lesions, and frailty were associated with increased ischemic risk in subgroup comparison. Abbreviations: CKD, chronic kidney disease (eGFR < 60 mL/min/1.73 m^2^); DES, drug-eluting stent; LM, left main coronary artery; LCX, left circumflex artery.

**Figure 5:**
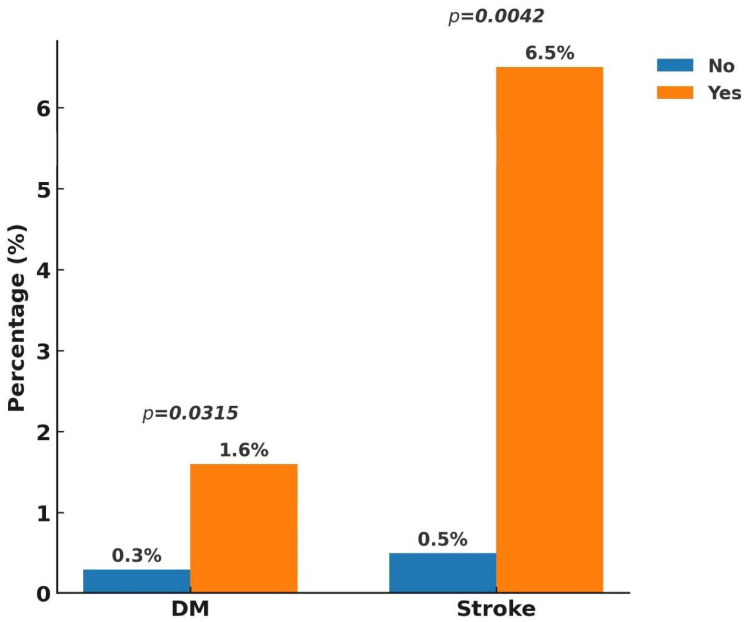
Subgroup analysis of one-year major bleeding. Major bleeding was defined as Bleeding Academic Research Consortium (BARC) types 3–5. A history of stroke and the presence of diabetes mellitus were associated with increased risk of major bleeding events. Abbreviations: DM, diabetes mellitus.

**Figure 6:**
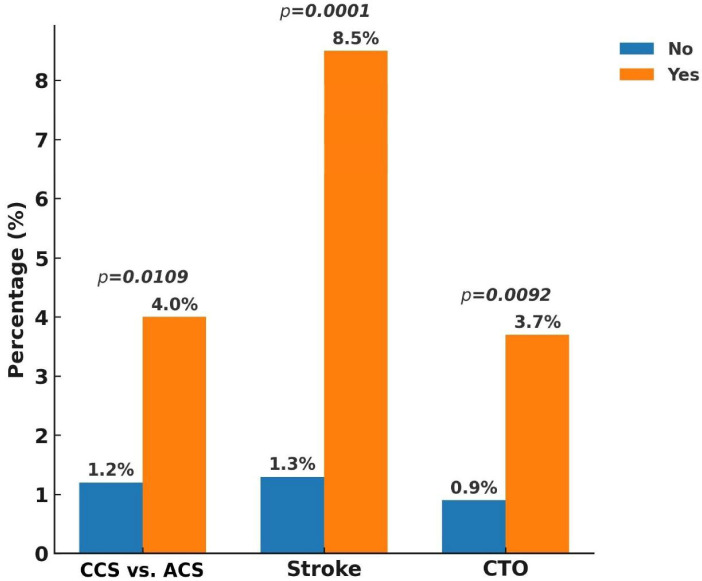
Subgroup analysis of one-year combined major and minor bleeding. Combined bleeding included BARC types 2–5. Bleeding events were more frequent among patients with CCS, prior stroke, and those undergoing CTO intervention. Abbreviations: BARC, Bleeding Academic Research Consortium; CCS, chronic coronary syndrome; CTO, chronic total occlusion.

At the one-year follow-up, the overall incidence of MACEs was 1.9%, with cardiovascular death occurring in 0.3%, non-fatal MI in 1.5%, and non-fatal stroke in 0.1%. When including unplanned revascularization, the composite ischemic event rate increased to 8.2%. Stent thrombosis was observed in 1.5% of patients, while all-cause mortality reached 1.3%. In terms of bleeding outcomes, major bleeding was recorded in 0.8%, whereas the overall rate of major plus minor bleeding was 1.7% (Figure 2). Subgroup analysis (Figure 3, Figure 4, Figure 5 and Figure 6) identified significant predictors associated with various clinical outcomes. HTN (*p* = 0.0130), total stent length ≥ 30 mm (*p* = 0.0454), and LCX as the target vessel (*p* = 0.0068) were significantly associated with MACEs. Composite ischemic outcomes (MACE plus unplanned revascularization) were significantly correlated with CKD (*p* = 0.0086), total stent length ≥ 30 mm (*p* = 0.0317), use of drug-eluting stents (DESs) (*p* = 0.0161), fragile patient group (*p* = 0.0086), LM lesions (*p* = 0.0034), and LCX lesions (*p* = 0.0114). Additionally, major bleeding was significantly associated with DM (*p* = 0.0315) and a history of stroke (*p* = 0.0042), while combined major and minor bleeding was significantly linked to CCS versus ACS diagnosis (*p* = 0.0109), stroke history (*p* = 0.0001), and CTO lesions (*p* = 0.0092). Other results from the subgroup analysis are presented in Supplementary Table S2.

**Figure 7:**
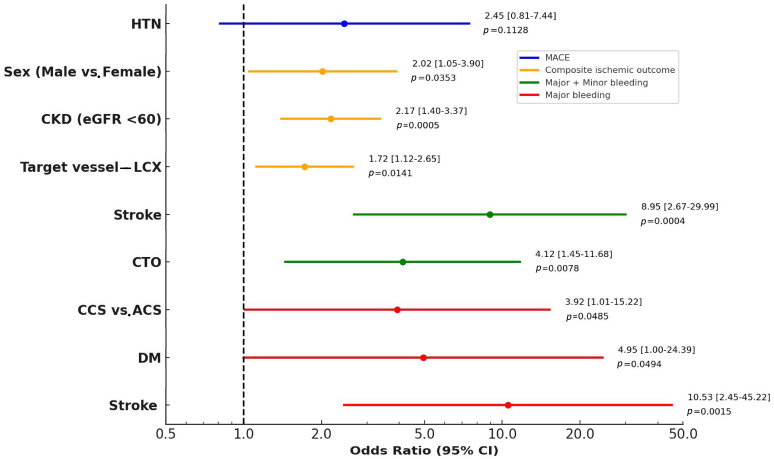
Multivariate logistic regression analysis of one-year clinical outcomes. Independent predictors of MACEs (cardiovascular death, non-fatal MI, and non-fatal stroke), composite ischemic events (MACE + unplanned revascularization), major bleeding (BARC 3–5), and combined bleeding (BARC 2–5) are presented as odds ratios (ORs) with 95% confidence intervals (CIs). Abbreviations: BARC, Bleeding Academic Research Consortium; CCS, chronic coronary syndrome; CKD, chronic kidney disease; CTO, chronic total occlusion; DES, drug-eluting stent; DM, diabetes mellitus; HTN, hypertension; LCX, left circumflex artery; LM, left main coronary artery; CI, confidence interval; OR, odds ratio.

Univariate and multivariate logistic regression analyses were performed to further identify independent predictors for each clinical outcome. Results from the univariate analysis are presented in Supplementary Table S3. In the multivariate model, HTN demonstrated a trend toward being an independent predictor of MACEs, although it did not reach statistical significance (OR 2.45, 95% CI 0.81–7.44, *p* = 0.1128). Independent predictors of composite ischemic outcomes included male sex (OR 2.02, 95% CI 1.05–3.90, *p* = 0.0353), CKD (OR 2.17, 95% CI 1.40–3.37, *p* = 0.0005), and involvement of the LCX as the target vessel (OR 1.72, 95% CI 1.12–2.65, *p* = 0.0141). For major plus minor bleeding events, stroke history (OR 8.95, 95% CI 2.67–29.99, *p* = 0.0004) and the presence of CTO lesions (OR 4.12, 95% CI 1.45–11.68, *p* = 0.0078) were identified as independent predictors. Regarding major bleeding alone, independent predictors included a diagnosis of CCS versus ACS (OR 3.92, 95% CI 1.01–15.22, *p* = 0.0485), DM (OR 4.95, 95% CI 1.00–24.39, *p* = 0.0494), and stroke history (OR 10.53, 95% CI 2.45–45.22, *p* = 0.0015) (Figure 7).

## 4. Discussion

In this real-world, multicenter registry study conducted across Taiwan, we evaluated the clinical effectiveness and safety of reduced-dose prasugrel (loading dose 20 mg, maintenance dose 3.75 mg) in patients with both ACS and CCS. The PROMISE-TW Registry enrolled 1167 patients, the majority with ACS and undergoing PCI. At one-year follow-up, the incidence of MACEs was low (1.9%), and the rate of composite ischemic events was 8.2%. Major bleeding events occurred in only 0.8%, and overall bleeding (major + minor) was 1.7%, suggesting an acceptable ischemic–bleeding balance. These results support the feasibility of using reduced-dose prasugrel in daily practice for the Taiwanese population, particularly in the ACS setting.

The 1-year MACE rate observed in our cohort (1.9%) was notably lower than that reported in the PRASFIT-ACS randomized trial (9.4%) despite both studies using an identical reduced-dose prasugrel regimen [6]. Furthermore, compared with the JAMIR-KAMIR registry analysis, which reported a 1-year MACE rate of 4.7% in East Asian AMI patients treated with adjusted-dose prasugrel, our findings suggest even more favorable outcomes in the Taiwanese population [13]. Notably, JAMIR-KAMIR also showed a major bleeding rate of 0.43% in the reduced-dose group compared to 1.71% in the standard-dose group, supporting the safety advantage of prasugrel dose adjustment in East Asian patients. The relatively low bleeding incidence in PROMISE-TW (0.8% major bleeding; 1.7% major + minor) supports the hypothesis of the “East Asian paradox,” wherein East Asian patients exhibit lower thrombotic but higher bleeding tendencies [14]. This pharmacodynamic profile, along with CYP2C19 polymorphisms prevalent in East Asians, necessitates region-specific strategies. The clinical adoption of low-dose prasugrel in Taiwan mirrors Japan’s approach and reflects growing consensus on tailored antiplatelet therapy for this population.

In Taiwan, several observational studies have also demonstrated consistent results with our findings. A recent single-center registry from Chang et al. reported a 1-year MACE rate (defined as cardiac mortality, non-fatal MI, the need for target lesion revascularization, non-fatal ischemic stroke, and stent thrombosis) of 7.1% and a major bleeding rate of 0.8% among 226 Taiwanese ACS patients receiving reduced-dose prasugrel [15]. Similarly, in the multicenter SWITCH study involving Taiwanese ACS patients, the 48-week incidence of MACEs was low (1.0%), and the major bleeding rate remained acceptable (2.0%) following a switch from clopidogrel to prasugrel [16]. Collectively, these results align closely with our study, suggesting that the effectiveness and safety of reduced-dose prasugrel are consistently observed across different Taiwanese populations and clinical contexts.

Importantly, our multivariate analysis revealed that patients with prior stroke, diabetes mellitus, and those undergoing CTO interventions had significantly higher bleeding risk. Specifically, a prior history of stroke increased the odds of major bleeding by more than 10-fold (OR 10.53, 95% CI 2.45–45.22), aligning with the result of subgroup analysis in the TRITON-TIMI 38 study [4] and current international guidelines. The 2023 ESC guidelines for the management of acute coronary syndromes explicitly contraindicate the use of prasugrel in patients with prior stroke due to elevated bleeding risk [17]. Our study thus reinforces the importance of guideline adherence in high-risk subgroups. In addition, although reduced-dose prasugrel has been approved for ACS in Taiwan since 2018, its use in CCS patients in our registry was off-label under current regulations. In this subgroup, ischemic events were infrequent, but we observed an elevated bleeding risk—especially among those with prior stroke, diabetes, or chronic total occlusion lesions—which warrants a cautious approach. Accordingly, these findings should be regarded as exploratory, and the primary applicability of our data remains within the ACS population. Further prospective, randomized studies are needed before considering broader off-label use in CCS and to inform any future regulatory or guideline updates on expanded prasugrel indications in Taiwan.

Subgroup analysis of ischemic endpoints revealed several key predictors associated with adverse outcomes. For MACEs, hypertension, stent length ≥ 30 mm, and LCX as the target vessel were significantly correlated with increased event rates. Notably, CKD (eGFR < 60 mL/min/1.73m^2^), long total stent length (≥30 mm), DES use, LCX and LM lesions, and frailty were significantly associated with increased ischemic risk. Furthermore, multivariate analysis demonstrated that male sex, CKD, and LCX as target vessel emerged as independent predictors for composite ischemic outcomes. These findings reflect the interplay between anatomical complexity, comorbid conditions, and treatment strategies. For example, patients undergoing PCI to LCX or LM often present with more technically demanding lesions, which may result in residual ischemia or higher risk of restenosis. Likewise, frail patients may receive modified therapies or shorter durations of DAPT, further contributing to suboptimal outcomes.

Another key observation in this study was the frequent switching between P2Y12 inhibitors in real-world settings. Over half of the patients had previously received either clopidogrel or ticagrelor before transitioning to prasugrel. The leading causes for switching were dyspnea (especially from ticagrelor), bleeding, and intolerance. These patterns reflect the complexity of antiplatelet decision making in clinical practice and highlight the need for practical algorithms to guide therapy adjustment based on side effects, genetic factors (e.g., CYP2C19 polymorphism), and patient risk profiles.

Lastly, our study was conducted exclusively in East Asian patients, among whom distinct pharmacogenomic variants (e.g., CYP2C19 and CYP3A4/5 polymorphisms) and prescribing habits (such as routine use of reduced-dose prasugrel) differ from those in Western and other non-East Asian regions. As a result, the safety and efficacy profiles we observed may not fully translate to populations with alternative metabolic phenotypes, baseline bleeding risks, or antiplatelet treatment paradigms. Future prospective international trials enrolling diverse ethnic cohorts will be necessary to confirm the generalizability of reduced-dose prasugrel across different geographic and genetic backgrounds.

## 5. Limitations

Several limitations must be acknowledged. First, this was a retrospective, observational study and thus subject to potential selection and reporting bias. Second, while the overall sample size was sizable, some important subgroups—such as CCS patients, those with prior stroke, or those receiving oral anticoagulants—were relatively small, limiting the statistical power for subgroup analysis. Third, although clinical outcomes were carefully adjudicated, the possibility of under-reporting events such as minor bleeding or asymptomatic restenosis cannot be excluded. Fourth, we acknowledge that the single-arm design of the PROMISE-TW registry—without a clopidogrel or ticagrelor comparator—precludes direct assessment of relative efficacy and safety. Consequently, our results should be considered hypothesis generating and interpreted with appropriate caution. Fifth, the major-bleeding model included only nine events and three covariates (EPV = 3, Supplementary Table S5); although LASSO selection confirmed prior stroke as the sole predictor, the low EPV necessitates cautious interpretation of these results. Lastly, because reduced-dose prasugrel is not currently approved for CCS in Taiwan, our findings in this population should be considered exploratory and hypothesis generating only. Future prospective, multicenter studies—ideally randomized—are needed to validate our findings and support potential label expansion or guideline refinement.

## 6. Conclusions

Our findings support the use of reduced-dose prasugrel in Taiwanese patients with ACS, demonstrating consistent ischemic protection and low bleeding rates in real-world practice. These results align with local and regional data, reinforcing its clinical utility in East Asian populations. However, the single-arm design and the off-label inclusion of CCS patients limit direct comparative interpretation and subgroup generalizability. Further research is warranted to confirm its safety in specific high-risk subgroups and to refine individualized antiplatelet strategies.

## Figures and Tables

**Table 1:** Baseline clinical and angiographic characteristics of the study population.

Characteristic	All N (=1167)
Age	64.6 (15.5)
>75	162 (13.9)
Male	948 (81.2)
ACS vs. CCS	
STEMI	336 (28.8)
NSTEMI	314 (26.9)
Unstable angina	268 (23.0)
Chronic stable phase after ACS	45 (3.9)
CCS	198 (17.0)
Concomitant anti-thrombotic agents during the index event	
Nil	185 (15.9)
Aspirin	951 (81.5)
Warfarin	3 (0.3)
DOAC	14 (1.2)
Aspirin + warfarin	2 (0.2)
Aspirin + DOAC	12 (1.0)
PCI	1060 (90.8)
Undergoing coronary stent implantation	941 (80.6)
Drug-eluting stent	813 (69.7)
Bare-metal stent	156 (13.4)
Complex PCI	819 (70.2)
Left main	97 (8.3)
True bifurcation lesion	400 (34.2)
CTO	187 (16.0)
Stenting longer than 30 mm	566 (48.5)
≥3 stents	123 (10.5)
Syntax score ≥ 33	73 (6.3)
Calcified plaque requiring atherectomy	105 (9.0)
Mechanical support	148 (12.7)
Vascular access	
Radial arteries	845 (72.4)
Femoral arteries	260 (22.3)
The target coronary arteries during PCI	
Left main	97 (8.3)
LAD	653 (56.0)
LCX	335 (28.7)
RCA	373 (32.0)
SVG	2 (0.2)
3-vessel CAD	326 (27.9)
Comorbidities	
Hypertension	709 (60.8)
Diabetes	446 (38.2)
Hyperlipidemia	320 (27.4)
Chronic kidney disease	222 (19.0)
Stroke	49 (4.2)
Fragile population	8 (0.7)

Continuous variables are presented as the mean ± standard deviation (SD) for normally distributed data and median (interquartile range, IQR) for non-normally distributed data; categorical variables are shown for the number of patients (% of total patients). Here, ACS = acute coronary syndrome; CCS = chronic coronary syndrome; STEMI = ST-segment elevation myocardial infarction; NSTEMI = non-ST-segment elevation myocardial infarction; DOAC = direct oral anticoagulant; PCI = percutaneous coronary intervention; CTO = chronic total occlusion; LAD = left anterior descending artery; LCX = left circumflex artery; RCA = right coronary artery; SVG = saphenous vein graft; and CAD = coronary artery disease.

## Data Availability

The original contributions presented in this study are included in the article/Supplementary Materials. Further inquiries can be directed to the corresponding author.

## Supplementary Materials

The following supporting information can be downloaded at: https://www.mdpi.com/article/10.3390/jcm14165791/s1. Supplementary Table S1: Study variables collected in the prasugrel registry; Supplementary Table S2: Subgroup analysis of one-year clinical outcomes; Supplementary Table S3: Univariate logistic regression analyses of independent predictors for each clinical outcome; Supplementary Table S4: Model Fit, Discrimination, and Multicollinearity Diagnostics for Logistic Regression Analysis; Supplementary Table S5: Number of events per variable (EPV) in multivariable logistic regression models for cardiovascular and bleeding outcomes; Supplementary Table S6: Timing of switch from prior P2Y12 inhibitors to prasugrel.
