# Supplementary material for: Clinical Effectiveness and Safety of Reduced-Dose Prasugrel in Asian Patients: The PROMISE-TW Registry
Source: J Clin Med. 2025 Aug 15;14(16):5791. doi: 10.3390/jcm14165791 (PMC12386672; doi:10.3390/jcm14165791)
Supplement: Supplementary file 1 [file jcm-14-05791-s001.zip › jcm-3794042-supplementary.pdf]

Supplementary Table S1. Study variables collected in the prasugrel registry

Category	Variables
**Inclusion criteria**	● Status while receiving prasugrel (ACS, chronic stable phase after ACS, or CCS)<br>● PCI or not<br>● Concomitant anti-thrombotic agents during the index event (Nil, aspirin, warfarin, NOAC, aspirin + warfarin, or aspirin + NOAC)<br>● Switch from other P2Y12 inhibitors and the reasons
**Baseline characteristics**	● Age<br>● Gender<br>● Height, weight, blood pressure, and BMI<br>● The timing of initially receiving prasugrel (hospitalization or outpatient clinics)<br>● Hospitalization length during the index event while receiving prasugrel<br>● Medical history:<br>Hypertension, diabetes, hypercholesterolemia, hyper-triglycemia, stroke, peripheral arterial occlusion disease, congestive heart failure, atrial fibrillation, chronic kidney disease (eGFR <60), liver cirrhosis, COPD, asthma

	• Laboratory data at the timing of initially receiving prasugrel:<br>BUN, Creatinine, eGFR, peak Troponin-I (if admission due to AMI), peak Troponin-T (if admission due to AMI), WBC, Hb, Platelet , Glucose AC, HbA1C, total-cholesertol level, triglyceride, HDL-C, LDL-C, hs-CRP, bilirubin, AST, ALT, Na, K, uric acid, and NT-pro BNP levels.
**Target coronary lesion(s)**	• Diseased vessel<br>• Syntax score<br>• Number of target lesions<br>• LM or non-LM bifurcation lesions<br>• CTO lesions<br>• Long (>30mm requiring stent) or multiple lesions
**Concomitant cardiac diseases, relevant cardiovascular conditions, and peri-procedure medications**	• Structural/valvular heart disease<br>• Previous coronary artery bypass surgery<br>• Cardiovascular surgeon consultant<br>• Left ventricular function by echocardiography<br>• Mechanical support: IABP, PCPS, ECMO, impella, LVAD, ventilator<br>• Intravenous medications during PCI: inotropic agents, diuretics, atropine, glycoprotein IIb/IIIa inhibitors<br>• DAPT at admission and discharge

**Intervention procedures**	• Thrombosuction<br>• Arterial access<br>• Image or functional devices: IVUS, OCT, FFR<br>• Total stent length<br>• Total stent number<br>• Stent sizes<br>• Stent types (BMS, DES, or BVS)<br>• Rotablation<br>• Total contrast medium amount<br>• Procedure time<br>• Complete revascularization
**In-hospital outcomes**	• Cardiovascular and all-cause mortality<br>• Recurrent myocardial infarction (MI)<br>• Stroke<br>• Angiographic and procedure results<br>• Acute kidney injury<br>• Unplanned revascularization<br>• Stent thrombosis<br>• Bleeding complications<br>• Other acute post-MI complications<br>• Echocardiographic data
**6 and 12 months clinical outcomes**	• Cardiovascular and all-cause mortality<br>• Recurrent MI and stent thrombosis<br>• Stroke<br>• Unplanned revascularization

	• Heart failure hospitalization<br>• Bleeding complications<br>• Echocardiographic data

Supplementary Table S2. Subgroup analysis of one-year clinical outcomes

MACE

	Status while receiving prasugrel				
	ACS		CCS		
	n	%	n	%	p value
No	899	97.93	197	99.49	0.231
Yes	19	2.07	1	0.51	

	Age(years)				
	0-75		>75		
	n	%	n	%	p value
No	781	97.87	157	96.91	0.399
Yes	17	2.13	5	3.09	

	Sex				
	female		male		
	n	%	n	%	p value
No	215	98.17	930	98.1	1
Yes	4	1.83	18	1.9	

	HTN				
	no		yes		
	n	%	n	%	p value
No	455	99.34	690	97.32	0.013
Yes	3	0.66	19	2.68	

	DM				
	no		yes		
	n	%	n	%	p value
No	706	97.92	439	98.43	0.533
Yes	15	2.08	7	1.57	

Composite ischemic outcome

	Status while receiving prasugrel				
	ACS		CCS		
	n	%	n	%	p value
No	839	91.39	187	94.44	0.153
Yes	79	8.61	11	5.56	

	Age(years)				
	0-75		>75		
	n	%	n	%	p value
No	734	91.98	146	90.12	0.436
Yes	64	8.02	16	9.88	

	Sex				
	female		male		
	n	%	n	%	p value
No	208	94.98	863	91.03	0.056
Yes	11	5.02	85	8.97	

	HTN				
	no		yes		
	n	%	n	%	p value
No	427	93.23	644	90.83	0.145
Yes	31	6.77	65	9.17	

	DM				
	no		yes		
	n	%	n	%	p value
No	665	92.23	406	91.03	0.468
Yes	56	7.77	40	8.97	

Major bleeding

	Status while receiving prasugrel				
	ACS		CCS		
	n	%	n	%	p value
No	907	99.45	190	97.94	0.056
Yes	5	0.55	4	2.06	

	Age(years)				
	0-75		>75		
	n	%	n	%	p value
No	787	99.37	157	98.13	0.137
Yes	5	0.63	3	1.88	

	Sex				
	female		male		
	n	%	n	%	p value
No	214	99.07	934	99.26	0.678
Yes	2	0.93	7	0.74	

	HTN				
	no		yes		
	n	%	n	%	p value
No	454	99.78	694	98.86	0.098
Yes	1	0.22	8	1.14	

	DM				
	no		yes		
	n	%	n	%	p value
No	713	99.72	435	98.42	0.032
Yes	2	0.28	7	1.58	

Major or minor bleeding

	Status while receiving prasugrel				
	ACS		CCS		
	n	%	n	%	p value
No	907	98.8	190	95.96	0.011
Yes	11	1.2	8	4.04	

	Age(years)				
	0-75		>75		
	n	%	n	%	p value
No	787	98.62	157	96.91	0.167
Yes	11	1.38	5	3.09	

	Sex				
	female		male		
	n	%	n	%	p value
No	214	97.72	934	98.52	0.378
Yes	5	2.28	14	1.48	

	HTN				
	no		yes		
	n	%	n	%	p value
No	454	99.13	694	97.88	0.102
Yes	4	0.87	15	2.12	

	DM				
	no		yes		
	n	%	n	%	p value
No	713	98.89	435	97.53	0.075
Yes	8	1.11	11	2.47	

Supplementary Table S2. (Continue)

MACE

CKD(eGFR <60)	no n	no %	yes n	yes %	p value
No	856	98.5	286	96.95	0.09
Yes	13	1.5	9	3.05	

CHF	no n	no %	yes n	yes %	p value
No	1006	98.24	133	97.08	0.317
Yes	18	1.76	4	2.92	

Stroke	no n	no %	yes n	yes %	p value
No	1098	98.21	45	95.74	0.224
Yes	20	1.79	2	4.26	

Bifurcation lesion	no n	no %	yes n	yes %	p value
No	778	97.86	367	98.66	0.353
Yes	17	2.14	5	1.34	

Target vessel -LM	no n	no %	yes n	yes %	p value
No	1052	98.32	93	95.88	0.103
Yes	18	1.68	4	4.12	

Composite ischemic outcome

CKD(eGFR <60)	no n	no %	yes n	yes %	p value
No	811	93.33	257	87.12	0.0008
Yes	58	6.67	38	12.88	

CHF	no n	no %	yes n	yes %	p value
No	939	91.7	126	91.97	0.914
Yes	85	8.3	11	8.03	

Stroke	no n	no %	yes n	yes %	p value
No	1025	91.68	44	93.62	1
Yes	93	8.32	3	6.38	

Bifurcation lesion	no n	no %	yes n	yes %	p value
No	731	91.95	340	91.4	0.749
Yes	64	8.05	32	8.6	

Target vessel -LM	no n	no %	yes n	yes %	p value
No	990	92.52	81	83.51	0.002
Yes	80	7.48	16	16.49	

Major bleeding

CKD(eGFR <60)	no n	no %	yes n	yes %	p value
No	856	99.3	289	98.97	0.7
Yes	6	0.7	3	1.03	

CHF	no n	no %	yes n	yes %	p value
No	1009	99.41	133	97.79	0.079
Yes	6	0.59	3	2.21	

Stroke	no n	no %	yes n	yes %	p value
No	1103	99.46	43	93.48	0.004
Yes	6	0.54	3	6.52	

Bifurcation lesion	no n	no %	yes n	yes %	p value
No	781	99.11	367	99.46	0.727
Yes	7	0.89	2	0.54	

Target vessel -LM	no n	no %	yes n	yes %	p value
No	1054	99.34	94	97.92	0.168
Yes	7	0.66	2	2.08	

Major or minor bleeding

CKD(eGFR <60)	no n	no %	yes n	yes %	p value
No	856	98.5	289	97.97	0.595
Yes	13	1.5	6	2.03	

CHF	no n	no %	yes n	yes %	p value
No	1009	98.54	133	97.08	0.268
Yes	15	1.46	4	2.92	

Stroke	no n	no %	yes n	yes %	p value
No	1103	98.66	43	91.49	1E-04
Yes	15	1.34	4	8.51	

Bifurcation lesion	no n	no %	yes n	yes %	p value
No	781	98.24	367	98.66	0.6
Yes	14	1.76	5	1.34	

Target vessel -LM	no n	no %	yes n	yes %	p value
No	1054	98.5	94	96.91	0.205
Yes	16	1.5	3	3.09	

Supplementary Table S2. (Continue)

MACE

	Target vessel -LAD				
	no		yes		
	n	%	n	%	p value
No	507	98.64	638	97.7	0.244
Yes	7	1.36	15	2.3	
	**Target vessel -LCX**				
	no		yes		
	n	%	n	%	p value
No	822	98.8	323	96.42	0.007
Yes	10	1.2	12	3.58	
	**Target vessel -RCA**				
	no		yes		
	n	%	n	%	p value
No	780	98.24	365	97.86	0.655
Yes	14	1.76	8	2.14	
	**CTO**				
	no		yes		
	n	%	n	%	p value
No	842	97.91	183	97.86	1
Yes	18	2.09	4	2.14	
	**Total stent length**				
	<30mm		>= 30mm		
	n	%	n	%	p value
No	307	99.35	609	97.44	0.045
Yes	2	0.65	16	2.56	

Composite ischemic outcome

	Target vessel -LAD				
	no		yes		
	n	%	n	%	p value
No	474	92.22	597	91.42	0.624
Yes	40	7.78	56	8.58	
	**Target vessel -LCX**				
	no		yes		
	n	%	n	%	p value
No	775	93.15	296	88.36	0.007
Yes	57	6.85	39	11.64	
	**Target vessel -RCA**				
	no		yes		
	n	%	n	%	p value
No	734	92.44	337	90.35	0.225
Yes	60	7.56	36	9.65	
	**CTO**				
	no		yes		
	n	%	n	%	p value
No	784	91.16	171	91.44	0.092
Yes	76	8.84	16	8.56	
	**Total stent length**				
	<30mm		>= 30mm		
	n	%	n	%	p value
No	294	95.15	567	90.72	0.018
Yes	15	4.85	58	9.28	

Major bleeding

	Target vessel -LAD				
	no		yes		
	n	%	n	%	p value
No	506	99.02	642	99.38	0.519
Yes	5	0.98	4	0.62	
	**Target vessel -LCX**				
	no		yes		
	n	%	n	%	p value
No	819	99.39	329	98.8	0.289
Yes	5	0.61	4	1.2	
	**Target vessel -RCA**				
	no		yes		
	n	%	n	%	p value
No	781	99.11	367	99.46	0.727
Yes	7	0.89	2	0.54	
	**CTO**				
	no		yes		
	n	%	n	%	p value
No	852	99.42	180	98.9	0.354
Yes	5	0.58	2	1.1	
	**Total stent length**				
	<30mm		>= 30mm		
	n	%	n	%	p value
No	305	99.35	616	99.35	1
Yes	2	0.65	4	0.65	

Major or minor bleeding

	Target vessel -LAD				
	no		yes		
	n	%	n	%	p value
No	506	98.44	642	98.32	1
Yes	8	1.56	11	1.68	
	**Target vessel -LCX**				
	no		yes		
	n	%	n	%	p value
No	819	98.44	329	98.21	0.78
Yes	13	1.56	6	1.79	
	**Target vessel -RCA**				
	no		yes		
	n	%	n	%	p value
No	781	98.36	367	98.39	0.971
Yes	13	1.64	6	1.61	
	**CTO**				
	no		yes		
	n	%	n	%	p value
No	852	99.07	180	96.26	0.009
Yes	8	0.93	7	3.74	
	**Total stent length**				
	<30mm		>= 30mm		
	n	%	n	%	p value
No	305	98.71	616	98.56	1
Yes	4	1.29	9	1.44	

Supplementary Table S2. (Continue)

MACE

Total stent number	<3 n	<3 %	>=3 n	>=3 %	p value
No	805	98.41	118	95.93	0.074
Yes	13	1.59	5	4.07	

Stent type-DES	no n	no %	yes n	yes %	p value
No	363	98.64	782	97.87	0.37
Yes	5	1.36	17	2.13	

Complex PCI	No n	No %	Yes n	Yes %	p value
No	344	98.85	801	97.8	0.228
Yes	4	1.15	18	2.2	

Single or combination therapy	Prasugrel SAPT n	Prasugrel SAPT %	Combination n	Combination %	p value
No	181	97.84	964	98.17	0.767
Yes	4	2.16	18	1.83	

Composite ischemic outcome

Total stent number	<3 n	<3 %	>=3 n	>=3 %	p value
No	754	92.18	113	91.87	0.906
Yes	64	7.82	10	8.13	

Stent type-DES	no n	no %	yes n	yes %	p value
No	329	89.4	742	92.87	0.045
Yes	39	10.6	57	7.13	

Complex PCI	No n	No %	Yes n	Yes %	p value
No	327	93.97	744	90.84	0.076
Yes	21	6.03	75	9.16	

Single or combination therapy	Prasugrel SAPT n	Prasugrel SAPT %	Combination n	Combination %	p value
No	169	91.35	902	91.85	0.82
Yes	16	8.65	80	8.15	

Major bleeding

Total stent number	<3 n	<3 %	>=3 n	>=3 %	p value
No	807	98.66	121	98.37	0.569
Yes	5	0.61	1	0.81	

Stent type-DES	no n	no %	yes n	yes %	p value
No	788	98.62	360	97.83	0.474
Yes	5	0.63	4	1.09	

Complex PCI	No n	No %	Yes n	Yes %	p value
No	341	98.55	807	99.51	0.137
Yes	5	1.45	4	0.49	

Single or combination therapy	Prasugrel SAPT n	Prasugrel SAPT %	Combination n	Combination %	p value
No	181	99.45	967	99.18	1
Yes	1	0.55	8	0.82	

Major or minor bleeding

Total stent number	<3 n	<3 %	>=3 n	>=3 %	p value
No	807	98.66	121	98.37	0.683
Yes	11	1.34	2	1.63	

Stent type-DES	no n	no %	yes n	yes %	p value
No	788	98.62	360	97.83	0.317
Yes	11	1.38	8	2.17	

Complex PCI	No n	No %	Yes n	Yes %	p value
No	341	97.99	807	98.53	0.5
Yes	7	2.01	12	1.47	

Single or combination therapy	Prasugrel SAPT n	Prasugrel SAPT %	Combination n	Combination %	p value
No	181	97.84	967	98.47	0.525
Yes	4	2.16	15	1.53	

Supplementary Table S3. Univariate logistic regression analyses of independent predictors for each clinical outcome

Variable	MACE					Composite ischemic outcome					Major bleeding					Major or minor bleeding				
	B	SE	Wald χ^2	p value	OR(95%CI)	B	SE	Wald χ^2	p value	OR(95%CI)	B	SE	Wald χ^2	p value	OR(95%CI)	B	SE	Wald χ^2	p value	OR(95%CI)
**Status while receiving prasugrel**																				
ACS vs CCS	-0.622	0.2357	6.9698	0.0083	0.29(0.11-0.73)	0.2352	0.1659	2.0101	0.1563	1.6(0.84-3.07)	-0.67	0.3378	3.9347	0.0473	0.26(0.07-0.98)	-0.622	0.2357	6.9698	0.0083	0.29(0.11-0.73)
**Switch from other P2Y12 inhibitors**																				
Plavix vs Nil	0.1363	0.3325	0.168	0.6819	1.65(0.55-4.95)	0.2494	0.1585	2.475	0.1157	0.79(0.45-1.38)	0.2269	0.4833	0.2204	0.6387	2.89(0.48-17.36)	0.1363	0.3325	0.168	0.6819	1.65(0.55-4.95)
Brilinta vs Nil	0.2272	0.3327	0.4666	0.4945	1.81(0.6-5.42)	-0.246	0.1815	1.8299	0.1761	1.29(0.8-2.07)	0.6056	0.4538	1.781	0.182	4.21(0.77-23.14)	0.2272	0.3327	0.4666	0.4945	1.81(0.6-5.42)
**Age(years)**																				
>75 vs 0-75	0.4119	0.2732	2.2735	0.1316	2.28(0.78-6.65)	0.1143	0.1469	0.6058	0.4364	1.26(0.71-2.24)	0.5506	0.3678	2.2414	0.1344	3.01(0.71-12.71)	0.4119	0.2732	2.2735	0.1316	2.28(0.78-6.65)
**SEX**																				
male vs female	-0.222	0.2632	0.7109	0.3992	0.64(0.23-1.8)	0.3109	0.1648	3.5597	0.0592	1.86(0.98-3.55)	-0.11	0.4027	0.0752	0.7839	0.8(0.17-3.89)	-0.222	0.2632	0.7109	0.3992	0.64(0.23-1.8)
**HTN**																				
Yes vs No	0.4487	0.283	2.5139	0.1128	2.45(0.81-7.44)	0.1647	0.1135	2.1051	0.1468	1.39(0.89-2.17)	0.8275	0.5312	2.427	0.1193	5.23(0.65-41.98)	0.4487	0.283	2.5139	0.1128	2.45(0.81-7.44)
**DM**																				
Yes vs No	0.4063	0.2343	3.0068	0.0829	2.25(0.9-5.65)	0.0785	0.1082	0.5262	0.4682	1.17(0.77-1.79)	0.8734	0.402	4.7198	0.0298	5.74(1.19-27.74)	0.4063	0.2343	3.0068	0.0829	2.25(0.9-5.65)
**CKD(eGFR <60)**																				
Yes vs No	0.1565	0.2491	0.3947	0.5298	1.37(0.52-3.63)	0.3632	0.1103	10.837	0.001	2.07(1.34-3.19)	0.1963	0.3552	0.3056	0.5804	1.48(0.37-5.96)	0.1565	0.2491	0.3947	0.5298	1.37(0.52-3.63)
**CHF**																				
Yes vs No	0.3524	0.2851	1.5277	0.2165	2.02(0.66-6.19)	-0.018	0.1671	0.0115	0.9147	0.97(0.5-1.86)	0.6667	0.3565	3.4973	0.0615	3.79(0.94-15.35)	0.3524	0.2851	1.5277	0.2165	2.02(0.66-6.19)
**Stroke**																				
Yes vs No	0.9614	0.2919	10.849	0.001	6.84(2.18-21.48)	-0.143	0.3032	0.2215	0.6379	0.75(0.23-2.47)	1.2757	0.362	12.42	0.0004	12.83(3.1-53.01)	0.9614	0.2919	10.849	0.001	6.84(2.18-21.48)
**Syntax score**																				
23-32 vs <=22	0.346	0.5346	0.419	0.5175	2.24(0.53-9.45)	0.1157	0.2103	0.3029	0.5821	1.42(0.82-2.48)	-0.216	0.7726	0.0783	0.7796	1.24(0.13-12.01)	0.346	0.5346	0.419	0.5175	2.24(0.53-9.45)
>=33 vs <=22	0.1123	0.7146	0.0247	0.8751	1.77(0.2-15.36)	0.1198	0.267	0.2012	0.6537	1.43(0.65-3.14)	0.6488	0.7743	0.7022	0.4021	2.95(0.3-28.72)	0.1123	0.7146	0.0247	0.8751	1.77(0.2-15.36)
**LM and diseased vessel**																				
1 vs LM	-0.606	0.4867	1.5512	0.213	0.31(0.07-1.38)	-0.197	0.1811	1.1769	0.278	0.68(0.35-1.3)	-0.838	0.8244	1.0341	0.3092	0.14(0.01-1.32)	-0.606	0.4867	1.5512	0.213	0.31(0.07-1.38)
2 vs LM	-0.025	0.4423	0.0032	0.9546	0.55(0.14-2.22)	-0.158	0.1964	0.648	0.4208	0.7(0.36-1.39)	0.1482	0.6558	0.051	0.8213	0.37(0.06-2.21)	-0.025	0.4423	0.0032	0.9546	0.55(0.14-2.22)
3 vs LM	0.0527	0.4425	0.0142	0.9053	0.59(0.15-2.4)	0.1609	0.185	0.7561	0.3846	0.97(0.5-1.87)	-0.467	0.8247	0.3208	0.5711	0.2(0.02-1.91)	0.0527	0.4425	0.0142	0.9053	0.59(0.15-2.4)
**Bifurcation lesion**																				
Yes vs No	-0.137	0.2624	0.2731	0.6012	0.76(0.27-2.13)	0.0362	0.1131	0.1022	0.7492	1.08(0.69-1.68)	-0.249	0.4021	0.3827	0.5362	0.61(0.13-2.94)	-0.137	0.2624	0.2731	0.6012	0.76(0.27-2.13)
**Target vessel -LM**																				
Yes vs No	0.3717	0.3191	1.357	0.2441	2.1(0.6-7.35)	0.447	0.1486	9.0479	0.0026	2.45(1.37-4.38)	0.5822	0.4045	2.0719	0.15	3.2(0.66-15.64)	0.3717	0.3191	1.357	0.2441	2.1(0.6-7.35)
**Target vessel -LAD**																				
Yes vs No	0.0402	0.2342	0.0295	0.8637	1.08(0.43-2.71)	0.0529	0.108	0.2398	0.6243	1.11(0.73-1.69)	-0.231	0.3367	0.469	0.4935	0.63(0.17-2.36)	0.0402	0.2342	0.0295	0.8637	1.08(0.43-2.71)
**Target vessel -LCX**																				
Yes vs No	0.0694	0.2489	0.0778	0.7803	1.15(0.43-3.05)	0.2915	0.1094	7.1031	0.0077	1.79(1.17-2.75)	0.3444	0.337	1.0447	0.3067	1.99(0.53-7.46)	0.0694	0.2489	0.0778	0.7803	1.15(0.43-3.05)
**Target vessel -RCA**																				
Yes vs No	-0.009	0.2488	0.0013	0.9713	0.98(0.37-2.61)	0.1338	0.1104	1.469	0.2255	1.31(0.85-2.02)	-0.249	0.4021	0.3827	0.5362	0.61(0.13-2.94)	-0.009	0.2488	0.0013	0.9713	0.98(0.37-2.61)
**CTO**																				
Yes vs No	0.7106	0.262	7.3555	0.0067	4.14(1.48-11.56)	-0.018	0.1438	0.0149	0.903	0.97(0.55-1.7)	0.3192	0.4203	0.5767	0.4476	1.89(0.36-9.84)	0.7106	0.262	7.3555	0.0067	4.14(1.48-11.57)

Supplementary Table S3. (Continue)

Variable	MACE					Composite ischemic outcome					Major bleeding					Major or minor bleeding				
	B	SE	Wald χ^2	p value	OR(95%CI)	B	SE	Wald χ^2	p value	OR(95%CI)	B	SE	Wald χ^2	p value	OR(95%CI)	B	SE	Wald χ^2	p value	OR(95%CI)
**Total stent length**																				
<30mm vs >=30mm	0.054	0.3025	0.0319	0.858	1.11(0.34-3.65)	0.3478	0.1492	5.4323	0.0198	2.01(1.12-3.6)	-0.005	0.4344	0.0001	0.991	0.99(0.18-5.44)	0.054	0.3025	0.0319	0.8583	1.11(0.34-3.65)
**Total stent number**																				
< 3 vs >=3	0.0964	0.3874	0.062	0.8034	1.21(0.27-5.54)	0.0209	0.1773	0.0138	0.9064	1.04(0.52-2.09)	0.1443	0.5498	0.0689	0.793	1.34(0.16-11.52)	0.0964	0.3874	0.062	0.8034	1.21(0.27-5.54)
**Stent type-BMS**																				
Yes vs No	-0.138	0.3762	0.1335	0.7148	0.76(0.17-3.32)	0.2601	0.136	3.6565	0.0559	1.68(0.99-2.87)	-0.107	0.5321	0.0406	0.8402	0.81(0.1-6.5)	-0.138	0.3762	0.1335	0.7148	0.76(0.17-3.32)
**Stent type-DES**																				
Yes vs No	-0.233	0.2345	0.9828	0.3215	0.63(0.25-1.58)	-0.217	0.1091	3.9558	0.0467	0.65(0.42-0.99)	-0.28	0.3369	0.6913	0.4057	0.57(0.15-2.14)	-0.233	0.2345	0.9828	0.3215	0.63(0.25-1.58)

Supplementary Table S4. Model fit, discrimination, and multicollinearity diagnostics for logistic regression analysis

Model Dependent Variable	Hosmer-Lemeshow test (p value)	C-statistic	Maximum VIF
Major adverse cardiovascular events	---	0.6305	1.0000
Composite ischemic events	0.8298	0.6332	1.0382
Combined major and minor Bleeding	---	0.7144	1.0000
Major bleeding	0.3138	0.7643	1.0063

Supplementary Table S5. Number of events per variable (EPV) in multivariable logistic regression models for cardiovascular and bleeding outcomes

**Dependent variable**	The number of events per variable (EPV)
**Major adverse cardiovascular events**	22 events /1 variable =22
**Composite ischemic events**	96 events /3 variable =32
**Combined major and minor bleeding**	19 events /2 variable ≈ 10
**Major bleeding***	9 events /3 variable =3

*we conducted LASSO penalized regression and compared AIC and C-statistic to the full logistic model; as no performance benefit was observed, we retained the full model

Supplementary Table S6. Timing of switch from prior P2Y12 inhibitors to prasugrel

**Timing of switch**	**Number (%)**
< 1 month	671 (63.5)
1-3 months	98 (9.3)
3-6 months	44 (4.2)
6-12 months	89 (8.4)
>12 months	155 (14.7)
